# Monitoring Resistance to Spinosad in the Melon Fly (*Bactrocera cucurbitae*) in Hawaii and Taiwan

**DOI:** 10.1100/2012/750576

**Published:** 2012-05-02

**Authors:** Ju-Chun Hsu, David S. Haymer, Ming-Yi Chou, Hai-Tung Feng, Hsaio-Han Chen, Yu-Bing Huang, Ronald F. L. Mau

**Affiliations:** ^1^Department of Entomology, National Taiwan University, Number 27, Lane 113, Sec. 4, Roosevelt Road, Taipei 10673, Taiwan; ^2^Department of Cell and Molecular Biology, University of Hawaii at Manoa, 1960 East-West Road, Biomed T511, Honolulu, HI 96822, USA; ^3^Department of Plant and Environmental Protection Sciences, University of Hawaii at Manoa, 3050 Maile Way Gilmore 310, Honolulu, HI 96822, USA; ^4^Taiwan Agricultural Chemicals and Toxic Substances Research Institute, Council of Agriculture, Wufong, Taichung 41358, Taiwan; ^5^Taiwan Agriculture Research Institute (COA), Wufong, Taichung 41362, Taiwan

## Abstract

Spinosad is a natural insecticide with desirable qualities, and it is widely used as an alternative to organophosphates for control of pests such as the melon fly, *Bactrocera cucurbitae* (Coquillett). To monitor the potential for development of resistance, information about the current levels of tolerance to spinosad in melon fly populations were established in this study. Spinosad tolerance bioassays were conducted using both topical applications and feeding methods on flies from field populations with extensive exposure to spinosad as well as from collections with little or no prior exposure. Increased levels of resistance were observed in flies from the field populations. Also, higher dosages were generally required to achieve specific levels of mortality using topical applications compared to the feeding method, but these levels were all lower than those used for many organophosphate-based food lures. Our information is important for maintaining effective programs for melon fly management using spinosad.

## 1. Introduction

Spinosad is a natural compound with insecticidal activity that has many properties considered to be highly desirable for insect control programs [1, 2]. This compound has been shown to be highly effective on a wide range of pest species, yet at the same time appear to have limited impact on nontarget organisms, including mammals, that may be exposed to it [2]. Moreover, spinosad is readily degradable by exposure to sunlight [2], thus minimizing any environmental burden that may occur as a result of widespread use. 

Spinosad was originally collected from a Caribbean island in 1985 [1], and the formulation that is currently the most widely used as an insecticide consists primarily of the A and D forms of this compound, both of which are naturally produced by the bacterial species *Saccharopolyspora spinosa*. Insecticide compounds based on spinosad have been extensively used as agents for control of insect pest species in the Diptera, Lepidoptera, Coleoptera, and Hymenoptera orders [3] among others. Within the Diptera, spinosad has been shown to be effective for control of Tephritid species within the *Ceratitis, Bactrocera, Rhagoletis, and Dacus genera* [2].

As with any compound used for control programs, however, one concern over such widespread use is the potential for resistance to this compound to arise either in laboratory and/or natural populations. Indeed, the history of both natural and artificial compounds used for insect control is replete with examples of resistance development even where much more highly toxic compounds such as DDT or malathion have been used [4, 5].

The *Bactrocera* species known as the melon fly, *B. cucurbitae* (Coquillett), causes significant economic damage to at least 81 different host plant species of cucurbits and melons. The wide distribution of this pest in Asia and Pacific areas cause quarantine concerns for several countries in these and other tropical, subtropical, and temperate regions of the world [6]. For most of the past forty years, organophosphate-(OP) compounds were the sole insecticides used to suppress this pest. Recently, due to growing environmental concerns raised over the use of OPs, alternatives such as spinosad have also been used [7, 8]. As part of a formulation known as GF-120 (Dow AgroSciences, Indianapolis, IN, USA), spinosad has been employed as part of an area-wide fruit fly pest management program (HAW-FLYPM) to control melon flies in Hawaii since 2002 [9, 10], and in central Taiwan since 2007.

In Hawaii the mild climate allows year round cucurbit crop production, and the populations of* B. cucurbitae* follow the crop production cycles and typically produce more than 10 generations per year [11, 12]. This means that control programs using spinosad or any insecticide, may subject the *B. cucurbitae* populations to intensive selection pressure over a relatively short-time frame. This also raises the specter of the potential for the rapid development of resistance. To monitor the possible development of resistance in populations to spinosad, it is essential to independently develop, for each pest, baseline information for the toxicity response and/or current levels of tolerance. This may be of great importance for this species because, of the major Tephritid pests found in areas such as Hawaii (*B. cucurbitae *along with* B. dorsalis* and *C. capitata*), the melon fly was the first to develop resistance to DDT as a control agent [13].

The objectives of this study include determination of the current levels of spinosad tolerance and/or susceptibility in *B*. *cucurbitae* using both the topical application and feeding methods of exposure, and assessments of current levels areas of tolerance in flies from populations in Hawaii and Taiwan where spinosad has been routinely applied anywhere for the past 2 to 6 years. Wild *B*. *cucurbitae* populations from Taiwan already showing resistance to the OPs fenthion and malathion have also been assayed to establish baseline tolerance levels prior to the use of spinosad, as well as to assess the potential for cross resistance to different control treatments in these populations.

## 2. Materials and Methods

### 2.1. Susceptible Laboratory Lines

The susceptible *B*. *cucurbitae *laboratory line used for the baseline study has been maintained at the USDA Tropical Fruit and Vegetable Research Laboratory in Honolulu, HI, USA under controlled environment conditions at  22 ± 3°C and 60–80% RH, for >300 generations without any contact to spinosad. Adults were kept in screen cages and supplied with a protein (hydrolysed yeast) sugar mixture (protein : sugar = 1 : 3) and water prior to testing [12]. The laboratory susceptible line from Taiwan was originally collected from Ilan, Taiwan, in 1998 and reared in an incubator at  24 ± 2°C, for >150 generations also without any exposure to spinosad.

### 2.2. Field-Collected *B. Cucurbitae* Populations

In Hawaii, three wild *B. cucurbitae* populations from the island of Oahu (Kunia, Kahuku, Ewa) and one from the island of Hawaii (Puna) were collected between June and July 2008 from infested host fruits, including zucchini (*Cucurbita pepo*), cucumber (*Cucumis sativus* L.), and papaya (*Carica papaya* L.) (Table 1). For the Taiwanese populations, infested sponge gourds (*Luffa aegyptiaca* Mill) and bitter gourds (*Momordica charantia* L.) were also collected from commercial farms in central Taiwan, Jhubei (Hsinchu), Erhshui (Changhwa), Puli (Nantou), Linnei (Yunlin), Dashe (Kaohsiung), and Jiouru (Pintung) (Table 1) between July and September 2007 to establish the field population cohorts. The infested fruits were incubated at 26 ± 2°C and 70 ± 5% r.h. for 7–14 days before pupae were collected. Emerging adults were supplied with cucumber and maintained for reproduction under the same conditions described for the laboratory line.

### 2.3. Bioassays

Bioassays were conducted with 3–5 d old F0 adults for field populations collected in Taiwan and for F1 adults from Hawaii populations. Topical and feeding bioassays were conducted to compare the spinosad susceptibility (LD_50_ or LC_50_ toxicities) between the wild populations and laboratory susceptible cohorts. Spinosad (Success 22.8% SC; Dow AgroSciences, Indianapolis, IN, USA) at 10 mg (a.i.)/mL was diluted with deionized water for tests in Hawaii. Analytical grade spinosad, fenthion, and malathion (Riedel-de Haën Co., Germany) were diluted by acetone at an initial concentration of 10 mg (a.i.)/mL to detect cross-resistance of the *B. cucurbitae* populations from Taiwan.

#### 2.3.1. Topical Application Assay

The procedure of topical application was described by Busvine [14]. Briefly, dilution series were prepared with acetone ranging from 0.3 to 100 ng/fly. Adults were anesthetized with carbondioxide, and 1 *μ*L of the tested solution was dropped onto the thoracic tergum [14]. The flies were then transferred to 250 mL plastic ice cream cups, provided with few drops of liquid food (sugar, yeast, and water, 4 : 1 : 5) [15]. Two replicates with a total of 40 flies (female : male = 1 : 1) were tested for each dose. Mortality in the treated adults was determined at 24 h posttreatment intervals for 72 h.

#### 2.3.2. Feeding Application Assay

The feeding application assay was as described by Hsu and Feng [16] and Chou et al. [17]. Spinosad dilutions ranging from 0.5 to 50 *μ*g/mL were prepared in dietary solutions containing 20% sugar and 5% peptone (wt/wt deionized water dilution). Cotton wicks (1 cm^3^) treated with approximately 0.2 mL solutions were prepared with four to seven different concentrations, with one group exposed to sugar solution as control for each tested cohorts. Twenty flies were exposed to the treated cotton wicks for 24 h in a 250 mL plastic ice cream cup. The treatment cotton wick was replaced by a new, insecticide-free wick during the 72 h observation. The accumulated mortality results were recorded at 24, 48, and 72 h after treatment. Two replicates with a total of 40 flies were tested for each dose. All treated flies were maintained in a room at a temperature of 24 ± 2°C and in a 12 : 12 h (L : D) photoperiod under fluorescent lamps.

### 2.4. Data Analysis

The posttreatment mortality data were subjected to probit analysis with POLO PC software [18] to obtain the LD_50_ (or LC_50_) and to compare the susceptibility slope of linear regression lines between cohorts. Treatments with 100% mortality were dropped from the data analysis in order to obtain the best fit linear regression response of mortality versus treatment. An *χ* ^2^ test was performed to assess how well the individual LC_50_ values observed in the bioassays agreed with the calculated linear regression lines [18]. The resistance ratio (RR) was calculated by the LD_50_ (or LC_50_) value of the wild population against the value of the laboratory line at the same posttreatment times.

Correlations were used to investigate possible cases of cross-resistance between the RR (wild fly/lab fly) of each of the three tested insecticides (spinosad, fenthion, and malathion) towards the wild fly populations collected from Taiwan using Excel- [19] based analyses.

## 3. Results

### 3.1. Topical Application Assays

The level of spinosad tolerance in flies from wild populations was measured by comparing the susceptibility of the field-collected melon flies to flies from the laboratory line (Table 2) using LD_50_ values and the 95% fiducial limits (FL). The LD_50_ values for the laboratory (susceptible) line ranged from a maximum of 5 ng/fly at 24 h after treatment to a minimum of 3.07 ng/fly at 72 h after treatment. The LD_50_ values for the various field populations from Hawaii were much broader in range ranging from a low of 1.86 ng/fly (Puna at 72 h) to a high of 16.7 ng/fly (Ewa at 48 h). Over the different time points, for the laboratory line the slopes of the dose-mortality regression lines decreased from a maximum of 3.27 ng/fly (24 h after treatment) to 3.12 ng/fly at 72 h. The same trend of decreasing values over time was also seen for flies from all of the Hawaiian populations sampled here, albeit again, generally beginning from higher values.

For the susceptible line, the LD_50_ values (and the 95% FL values) overlapped at the 24 versus 48 h and the 48 versus 72 h posttreatment points, but not for the 24 versus 72 h comparisons. For the wild collections, the highest LD_50_ and RR values (20.5 ng/fly; 5.28 times, resp.) for all of the posttreatment time points were found with the cohort collected from Ewa. In comparison to the laboratory susceptible line, the wild collections all differed in terms of their susceptibility to spinosad at all of the posttreatment times (based on LD_50_ and nonoverlap of 95% FL values), except for the collections from Puna and Kahuku at 72 h after treatment.

For the assays using topical applications of flies from the Taiwan populations, four of six collections exhibited lower susceptibility to spinosad compared with the LD_50_ value of the laboratory line (Table 3). Of these, the highest RR values were found for the Pintung collection followed by the Hsinchu collection. Among the Taiwanese populations, the slope values indicate that the flies from Nantou exhibited more heterogeneity in spinosad tolerance compared to any of the other tested populations.

### 3.2. Feeding Application Assays

The level of spinosad tolerance through feeding applications was also examined by comparing the level of susceptibility of the field-collected melon flies to that of the laboratory line using LC_50_ values and the 95% FL (Table 4). Here also, the LC_50_ values of the various strains declined as posttreatment time increased. The slopes of dose-mortality regression lines ranged from a maximum of 2.98 (wild collection from Pingtung at 72 h after treatment) to a minimum of 1.41 (wild collection from Kahuku at 72 h after treatment). The RR values increased as the posttreatment time increased, except for the collection from Puna, which had similar RR values between 48 h to 72 h after treatment.

For the laboratory susceptible line, the LC_50_ values ranged from a maximum of 3 *μ*g/mL at 24 h after treatment to a minimum of 0.65 *μ*g/mL at 72 h after treatment. The slopes dose-mortality regression lines for all three of these posttreatment time points were very similar. The 95% FL of the LC_50_ values overlapped only at the 48 and 72 h posttreatment time points.

In terms of the spinosad susceptibility in the wild populations, the cohort from Ewa showed the highest LC_50_ value (21.8 *μ*g/mL and RR up to 15.5 times) at all posttreatment times, followed by the population from Pingtung. With respect to the 95% FL values, only the flies from Puna (Hawaii) and Changhwa (Taiwan) showed LC_50_ values similar to the values found for the susceptible line at 24 h after treatment.

For all collections, the LC_50_ values at 48 h after treatment were similar to the values found at 72 h after treatment. However, the populations from laboratory, Kahuku and Ewa also exhibited significant difference in LC_50_ values between 24 h and 48 h after treatment. The resistance ratio was highest in the wild population collected from Ewa and lowest from Puna and Changhwa. With respect to different posttreatment time, the RRs rose 72 h after treatment, except in the collection from Puna.

### 3.3. Susceptibility of Field-Collected Flies to Spinosad, Malathion and Fenthion

**Figure 1:**
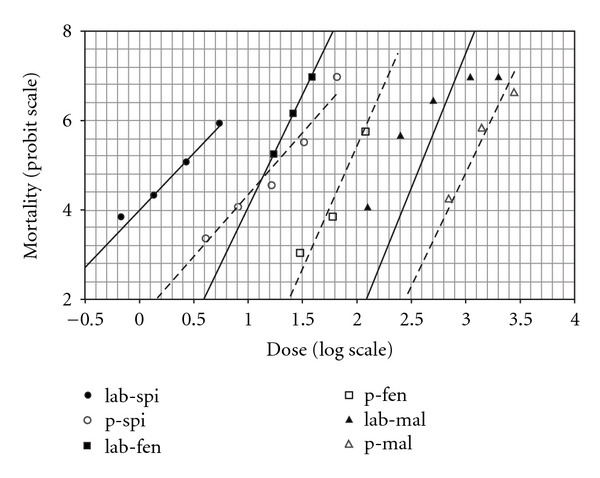
Dose-mortality regression line for spinosad, fenthion, and malathion by topical application in susceptible *B. cucurbitae* flies (solid line) and Pintung (dashed line) populations in Taiwan.

Table 5 lists the LD_50_ values for fenthion and malathion treatments for flies collected from the same locations given in Table 3 from Taiwan in 2007. The Pintung population showed the lowest susceptibility to all three tested insecticides, while flies from Yunlin showed the highest susceptibility to fenthion and spinosad.  Figure 1 shows that the slopes of the regression lines for the spinosad treatments were lower than those obtained for fenthion and malathion For the correlation analyses using the LD_50_ values of flies from various locations, that the only significant correlation seen was between the spinosad and fenthion treatments (*r* = 0.94, *P* < 0.05).

## 4. Discussion

The use of any agent, either natural or artificial, to control field populations of pest species requires information about several different factors. These include levels of toxicity using different exposure methods, current levels of tolerance in various strains or lines and the effects of prior exposure to either the same or different insecticidal treatments on these populations, and the efficacy of a natural control agent compared to more traditional methods of control which often involve the use of organophosphate insecticides. 

Regarding the issue of the relative toxicity using different exposure methods, our results show that in general the topical application method required higher dosages of spinosad to achieve LD_50_ values relative to the feeding application method. This is consistent with the fact that spinosad acts as a stomach poison, although spinosad it is activated by both contact and ingestion [2]. In addition, for some of the field populations, spinosad became more toxic by ingestion as the post treatment time increased. For example, the Ewa population showed significant increases in the resistance ratio at 48 and 72 h from the feeding bioassay. These values were also higher than those obtained from similar studies looking for possible delays in response to spinosad for other species such as *B. dorsalis *[16]. Except for the LD_50_ value at 72 h after treatment for the Puna collection, the LD_50_ or LC_50_ values from Puna are generally similar to the values of the Hawaii susceptible line. 

Questions relating to current levels of tolerance may be especially important for control agents such as spinosad because during the past decade field populations of some Lepidopteran and Bactrocera species, especially from areas with extensive use of spinosad, have been shown to develop resistance over relatively short periods of time [20–23]. Also, the control failures of diamondback moth (*Plutella xylostella*) recorded in field populations in Hawaii have been attributed to heavy selection pressure imposed by extensive use of spinosad [20]. Furthermore, laboratory studies have shown that selection for spinosad resistance can also be quite effective in species such as *Bactrocera dorsalis* [16], *Musca domestica*, and *Heliothis virescens* [24, 25]. Specifically for the case of *B. dorsalis*, it has been shown that up to 400-fold increased levels of resistance can develop after only eight generations of selection by topical application [16]. 

In terms of field applications, spinosad has been used since 2004 for control of *B. oleae* in California [26] and in Hawaii for control of both *B. cucurbitae* and *B. dorsalis* since 2000. In Hawaii, weekly GF-120 applications have been part of the HAW-FLYPM tactics to suppress melon fly damage in cucurbits and melon crops. Small area demonstrations began in 2000 at Kamuela (Hawaii), 2001 at Kula (Maui) and Central Oahu, and were adapted to an area wide approach used since 2002 [9, 10].^.^ However, prior to this study, no surveys had been conducted regarding baseline levels of spinosad tolerance in these populations or in this species. In our study, up to fifteenfold increased levels of spinosad resistance (by ingestion) was seen in some Hawaiian populations (e.g., Ewa). The RR values were similar to those reported for wild *B. oleae* populations in California [26], although higher than results seen for the wild *B. dorsalis* population in Hawaii [17]. This is all the more striking given that before 1990, despite heavy exposure to organophosphate insecticides, *B. cucurbitae *flies had shown no clear evidence of resistance under practical field conditions [27]. 

Overall, the resistance development seen in these studies of wild Tephritid flies is not as high as what has been found for wild populations of the species *Spodoptera exigua*, [21, 28] *P. xylostella*, [20] *Spodoptera litura*, [29] and the western flower thrips, *Frankliniella occidentalis* [30], in which up to 100-fold increased levels of resistance have been seen. 

Among the populations tested here from both Hawaii and Taiwan, the Ewa population of Oahu, Hawaii, exhibited the greatest tolerance to spinosad, followed by Kahuku and Kunia (also Hawaii), and Pingtung and Hsinchu (Taiwan). No difference in tolerance to spinosad was detected in the collections from Puna (Hawaii) or Nantou, Changhwa, Yunlin, and Kaohsiung (Taiwan). The reasons for the differing levels of tolerance of some areas of Hawaii and Taiwan may be different for each area. In Ewa, for example, spinosad is the main insecticide and applied in weekly doses year round in fields of zucchini, pumpkins, and melons, but others still used other insecticide for rotation. The year round application may have contributed to the higher tolerance compared to other more susceptible cohort populations. For Taiwan, by way of contrast, no spinosad had been applied for the control of melon flies prior to the year 2007. Among all the survey locations, melon flies from the Hsinchu population were the most tolerant to spinosad, but less so to OPs. The flies from Pingtung, however, were tolerant to both OPs and spinosad. Previous work had suggested that the carboxylesterase mechanism involved in OP resistance could promote cross-resistance to spinosad, but the reverse may not be true, specifically that the development of spinosad resistance does not automatically promote cross-resistance to OPs [16]. 

Also in Taiwan, fenthion and malathion have been the recommended insecticides for control of melon fruit fly control since the 1970s. In 2002, six Taiwanese populations (Hsinchu, Natou, Changhwa, Yunlin, Chiayi, Kaoshiung, Pingtung) were shown to have developed resistance to fenthion, malathion, and cross-resistance to fenthion and malathion (Hsu and Feng 2002). Our results show that the Taiwan collections showing higher tolerance to spinosad also showed higher tolerance to the organophosphate fenthion, and conversely the populations from these areas with the highest resistance to fenthion and malathion also exhibited the highest levels of resistance to spinosad. This indicates that it is likely that melon flies with resistance to organophosphate insecticides of fenthion or malathion also have higher potential to develop resistance to spinosad. Similar results were found in oriental fruit flies with high resistance to naled or malathion [16]. 

In conclusion, the field survey data analyzed here show that some spinosad resistance has already developed in wild populations of *B. cucurbitae*, likely as a result of extended commercial use in the fields. The resistance ratio seen in *B. cucurbitae *was, overall, moderately higher (10–13-fold) compared to the level seen in *B*. *oleae* populations in California [23], where spinosad has also been used extensively as a control agent. Also, even the relatively modest levels of increase seen in the *B. cucurbitae* populations contrasts with the fact that there is currently no evidence for spinosad resistance exhibited in wild populations of *B. dorsalis *[17], a species which is known to be capable of developing resistance to spinosad relatively easily after a short time of selection in the laboratory. This suggests that melon flies appear to be more sensitive to spinosad compared with other *Bactrocera* spp. [16, 23]. Using bioassays such as those described here to detect when changes in resistance reach a critical level, rotations of insecticides or incorporation of noninsecticide management practices can be implemented to avoid the development of further spinosad resistance in the melon fly.

## Figures and Tables

**Table 1:** Collections by location and global positioning data of wild *Bactrocera cucurbitae *populations tested for insecticide susceptibility in Hawaii and Taiwan.

Hawaii			Taiwan		
Location^1^	Latitude (N)	Longitude (W)	Location	Latitude (N)	Longitude (E)
Kunia (Oahu)	21°41′	158°04′	Jhubei (Hsinchu)	24°50′	129°59′
Kahuku (Oahu)	21°40′	157°57′	Puli (Nantou)	23°59′	120°57′
Ewa (Oahu)	21°20′	158°02′	Erhshui (Changhwa)	23°48′	120°37′
Puna (Hawaii)	19°42′	154°91′	Linnei (Yunlin)	23°45′	120°36′
			Dashe (Kaohsiung)	22°44′	120°21′
			Jiouru (Pintung)	22°44′	120°28′

^1^Date and host plant of the collections are as follows:

Kunia: 16-VIII-08, cucumber; Kahuku: 12-VIII-08, cucumber; Ewa: 22-VII-08, zucchini; Puna: 22-VIII-08, papaya.

Jhubei: 28-VIII-07, bitter gourd; Puli: 07-IX-07, sponge gourd; Erhshui: 14-IX-07, bitter gourd; Linnei: 22-VII-07, sponge gourd; Dashe: 09-VIII-07, sponge gourd; Jiouru: 21-IX-07, sponge gourd.

**Table 2:** Susceptibility and resistance to spinosad by topical application in *Bactrocera cucurbitae *flies (collected during August 2008) from wild populations and laboratory strains after 24, 48, and 72 h.

Collection	Regression parameters				RR^1^
	Slope ± SE	LD_50_ (ng/fly) (95% FL)^2^	*χ*²	*n*	
*24 h*					
Laboratory	3.12 ± 0.50	5.00 (4.02–6.07) c	3.80	280	
Kunia	2.70 ± 0.30	9.10 (7.56–11.04) e	3.40	219	1.82
Kahuku	2.18 ± 0.25	9.40 (6.89–13.5) e	4.77	260	1.88
Ewa	2.50 ± 0.26	20.5 (16.9–24.9) f	1.26	240	4.10
Puna	2.76 ± 0.35	3.96 (3.38–4.59) bc	0.87	249	0.79

*48 h*					
Laboratory	3.20 ± 0.47	3.16 (2.07–4.31) ab	4.52*	280	
Kunia	2.42 ± 0.27	6.44 (4.64–8.9) de	4.99*	219	2.04
Kahuku	2.53 ± 0.26	5.57 (4.64–6.67) d	2.63	260	1.82
Ewa	2.26 ± 0.24	16.7 (13.6–20.4) f	3.99	240	5.28
Puna	2.68 ± 0.48	2.60 (2.01–3.29) ab	0.53	249	0.82

*72 h*					
Laboratory	3.27 ± 0.47	3.07 (2.42–3.76) ab	3.10	280	
Kunia	2.52 ± 0.29	5.22 (4.28–6.32) c	2.81	219	1.70
Kahuku	2.64 ± 0.31	4.62 (3.15–6.28) bc	4.89	260	1.50
Ewa	2.56 ± 0.29	12.9 (7.80–19.5) ef	8.00*	240	4.19
Puna	1.89 ± 0.34	1.86 (1.17–2.43) a	2.46	249	0.61

*The asterisk (*) indicates a significant difference at *P* < 0.05 (*χ*² test) comparing the responses actually observed in the bioassay to the regression line from the probit analysis.

^1^The RR is given as the values of LD_50  of wild population_/LD_50  of laboratory strain_ to spinosad for the indicated post treatment time points.

^2^Within the LD column, different letters after the parentheses indicate significantly different LD_50_ values, as 95% FL did not overlap.

**Table 3:** Susceptibility of field populations of *B. cucurbitae* (collected during 2007) to spinosad by topical application at 24 h after treatment in Taiwan.

Location	Regression parameters						RR^a^
	*N*	Slope ± SE	LD_50_ (ng/fly) (95% FL)^1^		*χ*²	*n*	
Lab.	280	2.54 ± 0.31	2.42	(1.99–3.01) a	1.93	280	—
Hsinchu	200	3.36 ± 0.39	9.13	(7.73–11.0) c	2.92	240	3.77
Nantou	240	1.79 ± 0.22	4.59	(3.36–5.96) b	2.15	280	1.90
Changhwa	200	2.20 ± 0.31	4.63	(3.69–6.12) b	1.76	240	1.91
Yunlin	240	2.48 ± 0.39	2.71	(1.96–3.41) ab	1.51	240	1.12
Kaohsiung	240	2.35 ± 0.46	4.08	(2.36–5.53) ab	1.76	240	1.69
Pintung	200	2.77 ± 0.32	19.6	(13.9–28.6) d	3.71*	240	8.10

*The asterisk (*) indicates a significant difference at *P* < 0.05 (*χ*² test) comparing the responses actually observed in the bioassay to the regression line from the probit analysis.

^1^The RR is given as the values of LD_50  of wild population_/LD_50  of laboratory strain_ to spinosad for the indicated post treatment time points.

^2^Within the LD column, different letters after the parentheses indicate significantly different LD_50_ values, as 95% FL did not overlap.

**Table 4:** Susceptibility and resistance to spinosad by feeding application in *Bactrocera cucurbitae *flies (collected during August 2008 in Hawaii and from July to September 2007 in Taiwan) from wild populations and laboratory strains after 24, 48, and 72 h.

Collection	Regression parameters				RR^1^
	Slope ± SE	LC_50_ (*μ*g/mL) (95% FL)^2^	*χ*²	*N*	
*24 h*					
Laboratory	2.03 ± 0.26	3.00 (2.38–3.78) b	1.77	200	
Kahuku	1.86 ± 0.21	9.84 (5.93–16.9) c	8.29	240	3.28
Ewa	2.42 ± 0.39	21.8 (17.0–31.99) d	2.82	240	7.27
Puna	2.56 ± 0.30	4.68 (2.75–9.03) bc	11.27*	220	1.56
Changhwa	2.32 ± 0.32	3.07(2.36–3.80) b	2.34	200	1.32
Pingtung	2.60 ± 0.31	9.94(8.14–12.2) c	2.65	240	3.31

*48 h*					
Laboratory	2.02 ± 0.36	0.78 (0.46–1.07) a	1.23	200	
Kahuku	1.48 ± 0.22	2.53 (1.58–3.50) b	1.98	240	3.24
Ewa	2.28 ± 0.29	11.6 (9.39–15.0) c	3.70	240	14.9
Puna	2.28 ± 0.28	3.02 (2.05–4.35) b	4.35*	220	3.87
Changhwa	2.74 ± 0.38	2.78(2.19–3.37) b	0.82	200	3.56
Pingtung	2.86 ± 0.34	9.02(7.46–10.9) c	3.21	240	11.6

*72 h*					
Laboratory	1.83 ± 0.40	0.65 (0.26–1.03) a	2.65	200	
Kahuku	1.41 ± 0.22	2.14 (1.24–3.07) b	1.30	240	3.29
Ewa	2.29 ± 0.28	10.09 (6.36–19.4) cd	9.49*	240	15.5
Puna	2.11 ± 0.25	2.09 (1.12–3.57) b	8.20*	220	3.22
Changhwa	2.85 ± 0.40	2.59 (2.04–3.14) b	2.00	200	3.98
Pingtung	2.98 ± 0.35	8.67 (7.20–10.4) c	2.64	240	13.3

*The asterisk (*) indicates a significant difference at *P* < 0.05 (*χ*² test) comparing the responses actually observed in the bioassay to the regression line from the probit analysis.

^1^The RR is given as the values of LC_50  of wild population_/LC_50  of laboratory strain_ to spinosad for the indicated treatment time points.

^2^Within the LD column, different letters after the parentheses indicate significantly different LD_50_ values, as 95% FL did not overlap.

**Table 5:** Susceptibility of field populations of *B. cucurbitae*, collected during 2007 in Taiwan, to spinosad and other insecticides analyzed by topical application assay at 24 hr after treatment.

Insecticide and location	Regression parameters						RR^a^
	*N*	Slope ± SE	LD_50_ (95% FL)^1^		LD_90_ (95% FL)		
*Fenthion*							
Lab.	320	5.07 ± 1.12	15.4	(11.1–18.0) a	27.5	(24.0–35.0)	—
Hsinchu	200	4.13 ± 0.51	29.1	(25.0–33.6) b	59.4	(49.1–78.2)	1.89
Nantou	240	3.45 ± 0.49	30.8	(25.3–36.9) b	72.5	(57.5–104)	2.00
Changhwa	200	3.35 ± 0.39	32.9	(28.0–39.0) b	79.4	(63.3–110)	2.14
Yunlin	240	3.30 ± 0.42	21.0	(14.8–28.7) ab	51.3	(35.8–109)	1.36
Kaohsiung	152	3.56 ± 0.64	32.3	(27.1–40.2) b	73.9	(54.8–132)	2.10
Pintung	200	5.50 ± 0.74	88.3	(66.7–119) c	151	(114–303)	5.73

*Malathion*							
Lab.	280	6.07 ± 0.68	353	(322–387) a	574	(509–679)	—
Hsinchu	200	3.94 ± 0.48	367	(315–426) a	775	(638–1030)	1.04
Nantou	280	2.12 ± 0.26	508	(408–658) a	2050	(1390–3720)	1.44
Changhwa	200	3.27 ± 0.37	427	(361–505) a	1050	(840–1450)	1.21
Yunlin	240	3.40 ± 0.41	871	(639–1170) b	2080	(1470–4100)	2.47
Kaohsiung	200	4.41 ± 0.58	871	(755–1010) b	1700	(1410–2270)	2.47
Pintung	200	5.11 ± 0.68	1142	(998–1300) b	2040	(1720–2610)	3.24

^a^Resistance ratios (RR) toward insecticides are compared with the LD_50_ (95% FL) of laboratory line (lab.) in Taiwan.

^1^Within each insecticide, different letters after the parentheses indicate significantly different LD_50_ values, as 95% FL did not overlap.
